# Non-monotonic developmental trend of holistic processing in visual expertise: the case of Chinese character recognition

**DOI:** 10.1186/s41235-022-00389-3

**Published:** 2022-05-07

**Authors:** Ricky Van-yip Tso, Terry Kit-fong Au, Janet Hui-wen Hsiao

**Affiliations:** 1 Department of Psychology and Psychological Assessment & Clinical Research Unit, The Education University of Hong Kong, 10 Lo Ping Road, Tai Po, New Territories Hong Kong; 2 Department of Psychology, The University of Hong Kong, The Jockey Club Tower, Centennial Campus, Pokfulam Road, Pok Fu Lam, Hong Kong; 3 The State Key Laboratory of Brain and Cognitive Sciences, The University of Hong Kong, Pokfulam Road, Pok Fu Lam, Hong Kong

**Keywords:** Visual expertise, Holistic processing, Word recognition, Reading, Writing

## Abstract

Holistic processing has been identified as an expertise marker of face and object recognition. By contrast, reduced holistic processing is purportedly an expertise marker in recognising orthographic characters in Chinese. Does holistic processing increase or decrease in expertise development? Is orthographic recognition a domain-specific exception to all other kinds of recognition (e.g. face and objects)? In two studies, we examined the developmental trend of holistic processing in Chinese character recognition in Chinese and non-Chinese children, and its relationship with literacy abilities: Chinese first graders—with emergent Chinese literacy acquired in kindergarten—showed increased holistic processing perhaps as an inchoate expertise marker when compared with kindergartners and non-Chinese first graders; however, the holistic processing effect was reduced in higher-grade Chinese children. These results suggest a non-monotonic inverted U-shape trend of holistic processing in visual expertise development: An increase in holistic processing due to initial reading experience followed by a decrease in holistic processing due to literacy enhancement. This result marks the development of holistic and analytic processing skills, both of which can be essential for mastering visual recognition. This study is the first to investigate the developmental trend of holistic processing in Chinese character recognition using the composite paradigm.

## Significant statement

Holistic processing has been identified as an expertise marker of face and object recognition. Yet, reduced holistic processing is purportedly an expertise marker in recognising orthographic characters in Chinese which can be modulated by Chinese writing experiences. However, the conclusions from the existing research findings remained mixed on whether holistic processing increases or decreases in expertise development, and whether orthographic recognition is a domain-specific exception to all other kinds of recognition (e.g. face and objects in general). The results from this study fills in the above research gap by demonstrating that holistic processing indeed plays an important role in visual expertise across domains. For Chinese character recognition, holistic processing is an initial visual expertise marker that can be later reduced by further increase in Chinese literacy proficiency that requires more analytic processing. This non-monotonic trend of “rise then drop” in holistic processing marks the development of holistic and analytic processing skills, both of which can prove to be essential for visual expertise. This is the first use-inspired basic research study to investigate the developmental trend of holistic processing in Chinese character recognition using the composite paradigm.

## Introduction

**Fig. 1:**
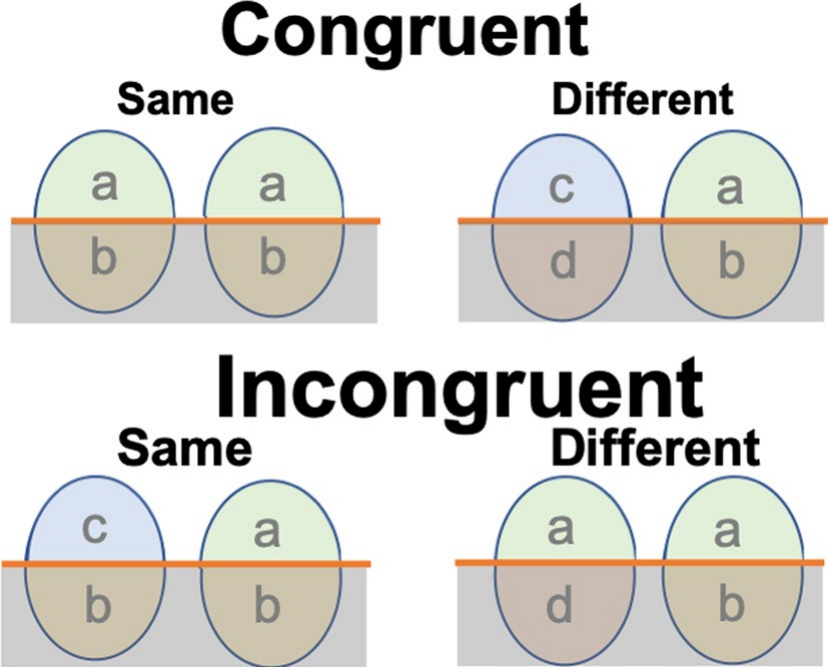
Complete composite paradigm to measure holistic processing for face stimuli. In each trial, participants were cued to attend to the top or bottom half of each stimulus pair and judged whether the attended halves were the same or different (attended halves encircled by the red rectangles in the figure). Holistic processing was demonstrated by a lower performance level in the incongruent condition than in the congruent condition, induced by the interference of the irrelevant/unattended halves

Holistic processing—the ability to process separate features jointly as a single whole unit—is a perceptual expertise marker for global processing in face and object recognition (Richler et al., 2011a, b, although some argued that holistic processing is specific to faces, e.g. McKone et al., 2007). Holistic processing of faces in children increases with age (e.g. Brace et al., 2001; Carey & Diamond, 1977; Schwarzer, 2002). Training adults to discriminate and individualise novel computer-synthesised objects with similar featural and configural properties also induced an increase in holistic processing (Gauthier et al., 1999; Wong et al., 2009). These findings suggest that holistic processing may be required for achieving expert-level visual object recognition, which automatises observers to rapidly discriminate and individualise visually similar objects within a category. Holistic processing in face recognition can be measured by inducing the *composite-face illusion* using the composite paradigm: e.g. the two identical top halves of a face are perceived as different when the bottom halves are from different face stimuli (Rossion, 2013). Composite-face illusion demonstrates obligatory attention to the whole face and failure to attend selectively only to individual face components (Richler et al., 2011a, b). Hence, holistic processing as assessed in the composite paradigm demonstrates the automaticitised tendency to process a visual object as a Gestalt (Pomerantz & Portillo, 2011), a type of configural processing as suggested by Maurer et al. (2002). Holistic processing provides spatial/configural information beyond individual features or configural information that probably allows experts to rapidly identify and individualise within-category objects (Richler et al., 2011a, b). See Fig. 1.

Although holistic processing is widely believed to be a general perceptual expertise marker across different domains that involve individualising within-category visual stimuli, several studies suggested the contrary. For example, expert Chinese character recognition is marked by reduced holistic processing (Chung et al., 2018; Liu et al., 2016), while novices (Hsiao & Cottrell, 2009; Tso et al., 2014) and Chinese readers with developmental dyslexia (Tso et al., 2020, 2021) recognised Chinese characters with a stronger holistic effect. These findings are also in contrast to the previously observed reduced holistic processing effect associated with face recognition difficulties in the clinical population, such as patients with prosopagnosia (Avidan et al., 2011) or autism (Gauthier et al., 2009). Hence, is orthographic recognition, or specifically, Chinese character recognition a domain-specific exception to all other kinds of recognition (e.g. face and objects)? This paper aims to contribute to the present literature on holistic processing by reviewing the existing work and examining the developmental trajectory of this perceptual phenomenon in Chinese character recognition in kindergarten and elementary-age children.

### Holistic processing and Chinese character recognition

Holistic processing may also play an important role in the recognition of visual words such as Chinese characters. In contrast to words in most alphabetic orthographies that are linear in structure and consist of letter series of varying length, the Chinese writing system is logographic—the configuration of Chinese characters is square and more homogenous than English words, and each character is a grapheme that, with a few exceptions, represents a morpheme (Shu, 2003; Wong & Gauthier, 2006). The basic units of a Chinese character are strokes; they combine to create more than a hundred basic stroke patterns—also known as radicals—in the Chinese writing system. These basic stroke patterns are put together to form characters; they are the smallest functional orthographic units for character recognition (Chen et al., 1996; Hsiao & Shillcock, 2006). The structure and configuration of facial features in a face follow a finite set of rules (i.e. the eyes typically has an exemplar of how it should be shaped and where it is on a face); similarly, the structure and character components within a Chinese character components are also predicted by a set of rules (i.e. orthography). A typical literate recognises more than 3000 individual characters regardless of variations in font (Hsiao & Lam, 2013; Hsiao & Cottrell, 2009), somewhat similar to recognition of faces being recognised individually regardless of variations in facial expressions (Hsiao & Cottrell, 2009; Wong & Gauthier, 2006). Indeed, the inversion effect, another common perceptual expertise marker observed in face perception for which faces are better recognised upright than inverted, has been widely demonstrated in experts during word recognition (Björnström et al., 2014; Kao et al., 2010). On the other hand, an expertise marker for Chinese character recognition seems to be reduced holistic processing, whereas for face recognition seems to be increased holistic processing (Hsiao & Cottrell, 2009).

Experienced Chinese readers use less holistic processing than novices in perceiving Chinese characters, perhaps because they are more sensitive to the constituent components (i.e. basic stroke patterns or radicals) within Chinese characters. They can readily ignore some configural information, such as exact distances between features, which are unimportant for character recognition (Ge et al., 2006). In contrast, the constituent components in a Chinese character may not readily pop out to novices, who do not distinguish among similar looking characters as readily as expert readers (Chen et al., 1996; Hsiao & Cottrell, 2009).

The reduced holistic effect observed in expert Chinese readers (Hsiao & Cottrell, 2009) could be due to writing experiences. Indeed, learning to recognise Chinese characters versus faces do differ: For example, while one needs not draw out faces seen day-to-day, a typical Chinese reader can recognise and write Chinese characters regularly. Unlike writing in an alphabetic orthography that requires knowing a few dozens of letters in the alphabet and their combination rules, writing in the Chinese logographic system for a typical literate requires writing characters by hand extensively (Tan et al., 2005; Tse et al., 2010). Thus, the reduced holistic processing observed in expert Chinese character recognition may be related to readers’ writing/sensorimotor experience rather than reading experience primarily.

Tso et al. (2014) tested holistic processing of Chinese characters on novices who could not read Chinese characters (Novices), Chinese literates who are not proficient in writing Chinese characters due to limited writing experience (i.e. Limited writers), and Chinese literates who could read and write in Chinese orthography proficiently (i.e. Writers). The Limited writers and the Writers did equally well in a word reading task, but the Limited writers performed much worse than the Writers in a dictation task (i.e. to recall and write down a Chinese word upon hearing it). Importantly, Limited writers showed increased holistic processing as compared with Novices, whereas Writers showed reduced holistic processing. That is, Writers perceived Chinese characters less holistically than Limited writers—this difference in holistic processing could be explained by writing performance, with reading performance variables statistically controlled for. These findings hint at a non-monotonic inverted U-shape learning trend in holistic processing: an increase in holistic processing of Chinese character recognition at the initial stages of learning to read in Chinese, with subsequent writing experiences reducing the holistic processing. The above speculations are consistent with Tso et al.’s (2020, 2021) findings that reading process in students with dyslexia in Chinese is marked by an increase in holistic processing in Chinese character recognition, when compared with their typically developing control. This increase in holistic processing is associated with poorer performance in writing rather than reading Chinese characters.

While recognition of faces and Chinese characters differs in important ways, there are some potentially important similarities. For example, although changes in spatial distances between components in Chinese characters should not affect the identity of the character, changes in distances between facial features likely affect face identity—however, configural changes in the spatial distances between components in a Chinese character still affect its recognition (Wong et al., 2019). Moreover, a right-hemispheric preference is identified for both face and Chinese character recognition (Hsiao & Cottrell, 2009; Hsiao & Liu, 2012; Liu et al., 2018; Chung et al., 2018; Tso et al., 2014); a decrease in right-hemispheric activities is found for face in prosopagnosics and for Chinese character recognition in dyslexics (Tso et al., 2020). Furthermore, while holistic processing generally increases with better face recognition for ordinary observers, reduced holistic processing has been found for artists with face drawing experiences, suggesting an effect of sensorimotor experience on holistic processing in face recognition (Zhou et al, 2012). Can analogous non-monotonic inverted U-shape trend in holistic processing be found in the developmental of expertise in Chinese character reading? More specifically, can reading experience lead to an initial increase in holistic processing of Chinese characters? Can Chinese writing experience then reduce holistic processing (i.e. lead to more analytic processing) of Chinese characters?

### The present studies

In Hong Kong, elementary school curriculum does not explicitly emphasise teaching Chinese character radicals (i.e. basic within-character stroke patterns). Nonetheless, children become more aware of the internal orthographic components in Chinese characters as they progress to higher grades (Ho et al., 2003). This could be explained by motor programming through extensive copying and writing Chinese characters, which are required activities in traditional Chinese language classes (Guan et al., 2011; Tan et al., 2005). Indeed, reading performance in Chinese is significantly predicted by writing and copying ability (Chan et al., 2006; McBride-Chang et al., 2011a, b; Tan et al., 2005; Tse et al., 2010). Learning to write has also been demonstrated experimentally to strengthen Chinese character recognition in adults learning Chinese as a second language (Guan et al., 2011) and plays an important role in shaping reading-specialised neural representations (James & Atwood, 2009; Longcamp et al., 2003; Siok et al., 2004). The development of sensory motor integration through writing practice may well be closely related to the development of reading skills, particularly for recognising Chinese characters.

Perhaps, expert proficiency in both reading and writing Chinese characters requires analytic processing of character components. Tso et al. (2020) found that adolescents with dyslexia in Chinese, who predictably did worse in Chinese character dictation and lexical decision than the typically developing controls, indeed showed stronger holistic processing in Chinese character recognition. This was also consistent with the findings on Limited writers (proficient readers with limited writing experience) reported in Tso et al. (2014), namely Limited writers’ stronger holistic processing being associated with poorer dictation performance. Selective attention to character components to form appropriate part-based representations seems important for typical Chinese literates proficient in both reading and writing.

Converging evidence suggests an important role of holistic processing in understanding the acquisition of Chinese character recognition skills (e.g. Hsiao & Cottrell, 2009; Liu et al., 2016) and the mechanism underlying character recognition difficulties in Chinese dyslexia (Tso et al., 2020). Although previous studies have hinted at a close relationship between children’s Chinese literacy development and holistic versus analytic processing, the underlying mechanism remains unclear. Here, we investigated the underlying perceptual mechanism by examining how holistic processing of Chinese characters is related to Chinese literacy development in kindergarten and elementary school children. Previous research suggested that reduction in holistic processing of Chinese characters marks increasing expertise in character recognition, and this reduction in holistic processing is related to the readers’ writing performance (Tso et al., 2014, 2020, 2021). Hence, Study 1 set out to examine the hypothesis that: (1) an initial increase in holistic processing may be necessary for kindergarteners to recognise and individualise Chinese characters because early print vocabulary includes characters that can be quite complex internally (Liu et al., 2016; Tso et al, 2014), and (2) mainstream elementary-school children’s holistic processing of Chinese characters decreases as they progress to higher grades.

In Study 2, to see if any observed holistic processing effect is indeed due to Chinese literacy development rather than general cognitive development, we examined holistic processing of Chinese characters in elementary school children learning Chinese as a non-native language (non-Chinese-speaking (NCS)) as a comparison group. Specifically, we used NCS first graders (who had far less experience with Chinese characters than the Chinese-speaking (CS) first graders with Chinese as native language in Study 1) but had comparable general cognitive development (e.g. receiving the same first-grade curriculum although in different languages) as a comparison/novice group. This allowed us to examine whether Chinese first graders exhibited stronger or weaker holistic processing as compared with first-grade NCS novice readers for Chinese. Any measurable differences in holistic processing effects between Chinese and NCS children would suggest an influential role of Chinese literacy development on holistic processing of Chinese characters, rather than on general cognitive development. This paper is also the first to investigate the role of holistic processing in the development of Chinese literacy.

## Study 1

In this study, we investigated whether children in upper elementary grades (who had better Chinese literacy) perceived characters less holistically (i.e. more analytically) than children in lower elementary grades. The decreasing trend constitutes the right arm of the hypothesised non-monotonic inverted U-shaped developmental trend in holistic processing for Chinese characters. The participants attended an elementary school using Chinese as the language of instruction for all classes except English lessons. We also assessed their Chinese reading and writing performance to examine how improvement in Chinese literacy may be related to children’s holistic processing of Chinese characters.

We also recruited Chinese-speaking kindergartners in the final year of a local three-year kindergarten programme and compared their holistic processing of Chinese characters with the elementary school children. Based on the high holistic processing level of adult Limited writers who could read but not write in Chinese proficiently, Tso et al. (2014) predicted an increase in holistic processing in emergent readers of Chinese characters than complete novices. Kindergartners in Hong Kong offer a good test case. They have initial exposure to reading instructions in Chinese, but literacy demand is minimal in the 3-year kindergarten programme (roughly from age 3 to 5; see Curriculum Development Council, 2007). The increasing literacy demand in the transition from kindergarten to elementary school may require young children to expand their mental lexicon of Chinese characters to accommodate and individualise the increasing number of new words. We hypothesised an increase in holistic processing in emergent Chinese readers from kindergarten to first grade—a rise in holistic processing constituting the left arm of the hypothesised non-monotonic U-shape developmental trend.

### Methods

Ethical Approval was granted by the Human Research Ethics Committee of the authors’ universities for this study. Legal guardian informed consent and child participant assent were obtained prior to data collection.

#### Participants

First-, third-, and fifth-grade Chinese children from an elementary school in Hong Kong participated in this study. Altogether, 30 first graders (mean age = 5.88 years, SE = 0.051), 30 third graders (mean age = 7.90, SE = 0.056), and 30 fifth graders (mean age = 9.89, SE = 0.047) participated in this study.^1^ We also recruited 30 final-year Chinese kindergartners (K3; mean age = 4.86 years, SE = 0.149) from a kindergarten in Hong Kong. They were all native Cantonese-Chinese speakers and receiving regular Chinese language curriculum at school. All of them were reported by their parents or guardians to have normal or corrected-to-normal vision. The design of the study is a group comparison in order to observe the patterns across grades. Hence, Grades 1, 3, and 5 were chosen to allow us to see if there is a significant pattern across grades, as differences between consecutive grades (e.g. Grades 1 vs. 2) may be too subtle to reveal statistical differences (see Davies et al., 2017; Guan & Fraundorf, 2020; Guan et al., 2020).

#### Materials and procedures

##### Reading and writing assessment

The children were administered Chinese literacy assessments. In the *word naming* task, children were presented with 30 two-character words, one at a time, and instructed to read them aloud. The words were arranged from high to low frequency (based on a local data corpus: Leung & Lee, 2001). Each trial started with a 500-ms central fixation cross, followed by the character presentation. The screen turned blank after a child had responded, and the experimenter pressed a button to record the accuracy and to start the next trial.

The *word dictation* task measured children’s ability to recall and write Chinese words. Children wrote down 30 two-character words (the same words used in the word naming task) as quickly and as accurately as possible when they heard each word spoken in a female voice presented by a computer. Two-character words were used instead of single characters to reduce ambiguity due to the many homophonic characters in the Chinese lexicon. Each trial started with the words “Get ready” on the screen for 500 ms. After hearing the stimulus word, participants wrote down the word. After they had finished writing, the experimenter pressed a button to indicate accuracy and to display the next word. Accuracy rate was recorded.

Note that the same materials were used to measure literacy for a controlled and fair measurement across grades. Also, the stimuli used in the dictation task were the same as those used in the word naming task for a cleaner comparison of dictation and naming performance. The order of naming and dictation tasks was counterbalanced across participants. Moreover, participants were not given the answers to any of the literacy tasks, so that their performance in subsequent tasks would not be affected. The 30 two-character words chosen were from the corpus of commonly used words by primary school students (Leung & Lee, 2001) and had been pretested to avoid both floor and ceiling effects across grades for both the dictation and word naming tasks. Note that the literacy tests were not administered to the K3 children due to a floor effect in our pilot and to avoid over testing.

##### Rapid automatised naming

Because general symbol processing speed is related to reading ability in children (Ho & Lai, 1999; Wolf & Bowers, 1999), we used a computerised rapid automatised naming (RAN) task to measure symbol processing speed (Denckla & Rudel, 1974). The digits 1–9 were presented one at a time for 4 times in a random order on the computer screen. Each trial started with a 500-ms central fixation cross, followed by the digit presentation. The screen turned blank after the naming response, and the experimenter pressed a button to record the accuracy and to start the next trial. RAN was measured as the response time (ms) between the stimulus onset and the onset of naming, detected by a voice key with a microphone.

##### Holistic processing

**Fig. 2:**
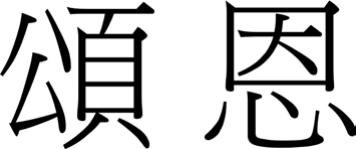
Examples of Chinese characters with a left–right configuration (left) and a top–bottom configuration (right)

**Fig. 3:**
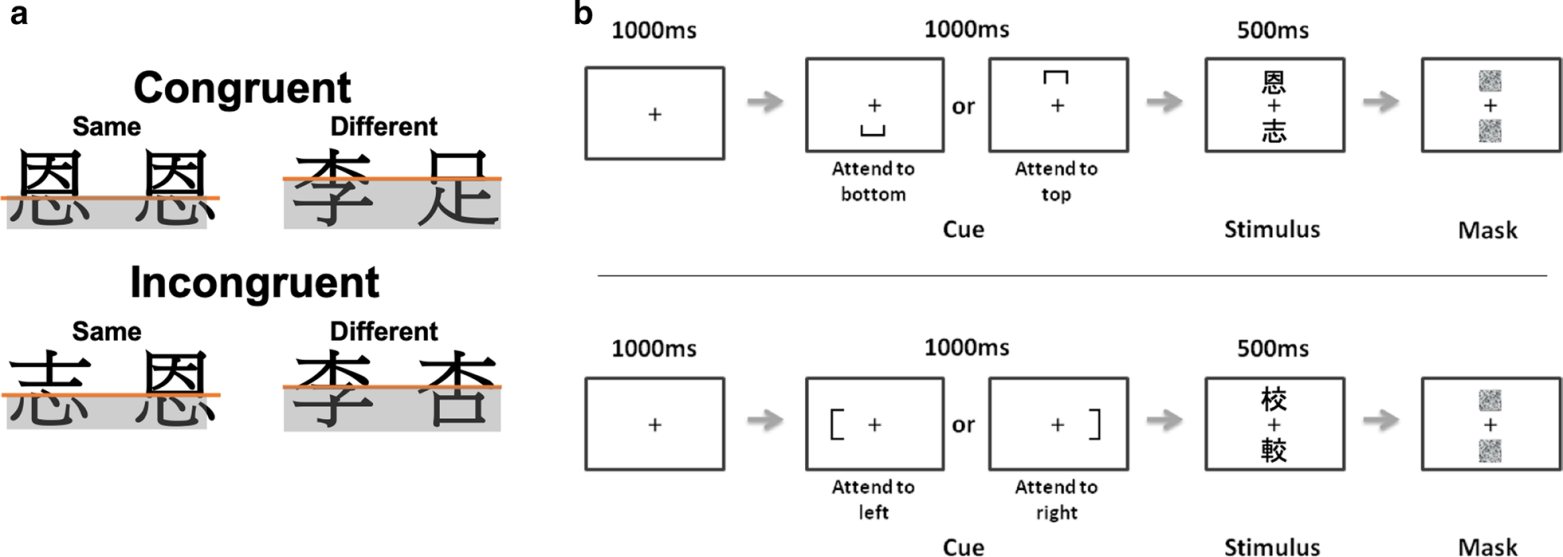
Illustration of stimulus pairs in the complete composite paradigm and trial sequences. In (**a**), it shows the four conditions used in the paradigm; the attended components are shaded in grey. In (**b**), a 1000 ms central fixation cross precedes each trial, followed by a cue either below or above the cross, or to the left or right of the cross, to indicate which halves (top or bottom/left or right) of the characters participants should attend to in the subsequent display. The complete composite paradigm (Gauthier & Bukach, 2007) was adopted so that in congruent trials, the attended and irrelevant/unattended halves corresponded to the same response (i.e. both were the same or both were different); by contrast, in incongruent trials, the attended and irrelevant/unattended halves would correspond to different judgments (e.g. the top halves were the same, while the bottom halves were different). We adopted this paradigm to avoid response biases that may occur in the partial composite design where the irrelevant/unattended halves are always different (Richler et al., 2011; Robbins & McKone, 2007)

To assess holistic processing, we adopted the complete composite paradigm as in Hsiao and Cottrell (2009) and Tso et al. (2014). One hundred and sixty pairs of medium- to high-frequency Chinese characters (Ho & Kwan, 2001) in Ming font were chosen, 80 pairs had a top–bottom configuration, and 80 pairs had a left–right configuration (Fig. 2). In each trial, children were presented with two characters and instructed to attend to only half (either top or bottom for top–bottom characters; either left or right for left–right characters) of each character and judge whether they were the same or different. Forty pairs (twenty top–bottom and twenty left–right character pairs) were presented in each of the four conditions (Fig. 3a): *same in congruent trials, different in congruent trials, same in incongruent trials, and different in incongruent trials*. The characters in all the conditions were matched in stroke number and frequency. A practice session with graphical instructions and 40 practice trials was administered with each child prior to this test to ensure understanding of the task. Formulation of the character stimuli used in the composite paradigm was independent of the literacy tests.

Note that in the partial composite paradigm, a misaligned condition must be administered (i.e. the top halves and bottom halves of the stimuli are misaligned from each other; e.g. Robbins & McKone, 2007). By contrast, in the complete composite design without an optional misaligned condition, holistic processing can be indicated by the performance difference between the congruent and incongruent trials (Gauthier & Bukach, 2007; Hsiao & Cottrell, 2009; Tso et al., 2014). One advantage of including a misaligned condition in the complete composite paradigm is to rule out the possibility that the congruency effect observed in the aligned condition is due to difficulty in response inhibition rather than selective attention. Hsiao and Cottrell (2009), Tso et al. (2020), and Tso et al. (2021) showed that the congruency effect observed in Chinese character processing disappeared when character halves were misaligned, suggesting that the effect was due to difficulty in selective attention. However, due to procedural and ethical constraints, a misaligned condition was not administered in the current studies to avoid repeated and prolonged testing for the children.

In each trial, after 1000 ms central fixation, children were cued with a symbol that indicated which half of each character they should attend to. The pair of characters was then presented, with one above and one below the initial fixation point, followed by a mask. During the 500-ms presentation time, children looked at each character and responded as quickly and accurately as possible by pressing corresponding buttons to judge if the relevant character parts were the same or different (Fig. 3b). Accuracy was recorded. We measured participants’ discrimination sensitivity *A*′ as:

$$A{\prime } = 0.5 + \left[ {{\text{sign(}}H - F{)}\frac{{{(}H - F{)}^{2} + \left| {H - F} \right|}}{{4\max {(}H,F{)} - 4HF}}} \right]$$

*H* and *F* are the hit rate and false alarm rate, respectively. *A*′ is a bias-free nonparametric measure of sensitivity, suggested to be in general more accurate than *d*′ (Donaldson, 1993); we did not use *d*′ as a precaution because response biases may affect its measurement when assumptions of normality and homogeneity of variance are not met (Stanislaw & Todorov, 1999). Unlike *d*′, it is possible to compute *A*′ even when zero responses are present in cells. An *A*′ of 0.5 denotes random chance, while 1.0 suggests perfect performance. The complete composite paradigm measures holistic processing by the performance difference between congruent and incongruent trials; the score difference of *A*′ between congruent and incongruent trials is also represented as Holistic *A*′ to give a clearer representation of holistic processing—a more positive value indicates a stronger holistic processing effect.

The length of each task was around 10–15 min. All children completed the literacy tasks in one session and the holistic processing tasks in another. All the tasks were administered in a randomised order. These experiments were all conducted using E-prime v2.0 (Psychology Software Tools, Pittsburgh, PA).

## Results

### Chinese reading and writing proficiency

One-way ANOVAs revealed grade-related increases in word-naming accuracy, *F*(2, 87) = 51.649, *p* < 0.001, *η*_p_^2^ = 0.543, dictation accuracy, *F*(2, 87) = 100.305, *p* < 0.001, *η*_p_^2^ = 0.698, as well as increase in rapid naming speed, *F*(2, 87) = 7.684, *p* = 0.001, *η*_p_^2^ = 0.150. As expected, the children had better Chinese reading and writing proficiency—giving more accurate and faster responses—in higher grades (Table 1).

**Table 1:** Means of RAN, reading, writing, and performance in first, third, and fifth graders

	Grade 1	Grade 3	Grade 5
	Mean (SD)		
Word-naming accuracy	0.47 (0.29)	0.87 (0.13)	0.94 (0.09)
Dictation accuracy	0.11 (0.06)	0.39 (0.20)	0.71 (0.20)
RAN (ms)	708 (204)	606 (118)	550 (139)

A 2 × 3 mixed-design ANOVA with literacy measures (word-naming vs. dictation) × grade (grades 1 vs. 3 vs. 5) revealed a main effect for grade, *F*(2, 87) = 110.026, *p* < 0.001, *η*_p_^2^ = 0.717, a main effect of literacy, *F*(1, 87) = 232.182, *p* < 0.001, *η*_p_^2^ = 0.727, and a significant literacy × grade interaction, *F*(2, 87) = 9.043, *p* < 0.001, *η*_p_^2^ = 0.172. Post hoc analyses (Bonferroni corrections: *α* ≤ 0.044) revealed that fifth graders named and wrote words more accurately than third- [*t*(58) = 2.476, *p* = 0.016, *d* = 0.64, and *t*(58) = 6.210, *p* < 0.001, *d* = 1.60, respectively] and first graders [*t*(58) = 8.380, *p* < 0.001, *d* = 2.16, and *t*(58) = 16.212, *p* < 0.001, *d* = 4.19, respectively], while third graders named and wrote words more accurately than first graders, [*t*(58) = 6.773, *p* < 0.001, *d* = 1.75 and *t*(58) = 7.506, *p* < 0.001, *d* = 1.94, respectively]. Word-naming accuracy was higher than dictation accuracy across all grades, *t*(89) = 14.023, *p* < 0.001, *d* = 1.48. To further understand the literacy × grade interaction, post hoc *t* tests were conducted on the score difference between the word-naming and dictation tasks (Bonferroni corrections: *α* ≤ 0.047): While this naming–writing discrepancy between first and third graders was comparable with only a marginal effect, *t*(58) = 1.913, *p* = 0.063, fifth graders had a small naming–writing discrepancy than third and first graders, *t*(58) = 2.033, *p* = 0.043, *d* = 0.52, and *t*(58) = 5.219, *p* < 0.001, *d* = 1.35. This shows that as the children progressed to upper grades, the discrepancy between their word-naming and writing performances narrows, coinciding with an increase in overall literacy performance.

### Holistic processing

**Fig. 4:**
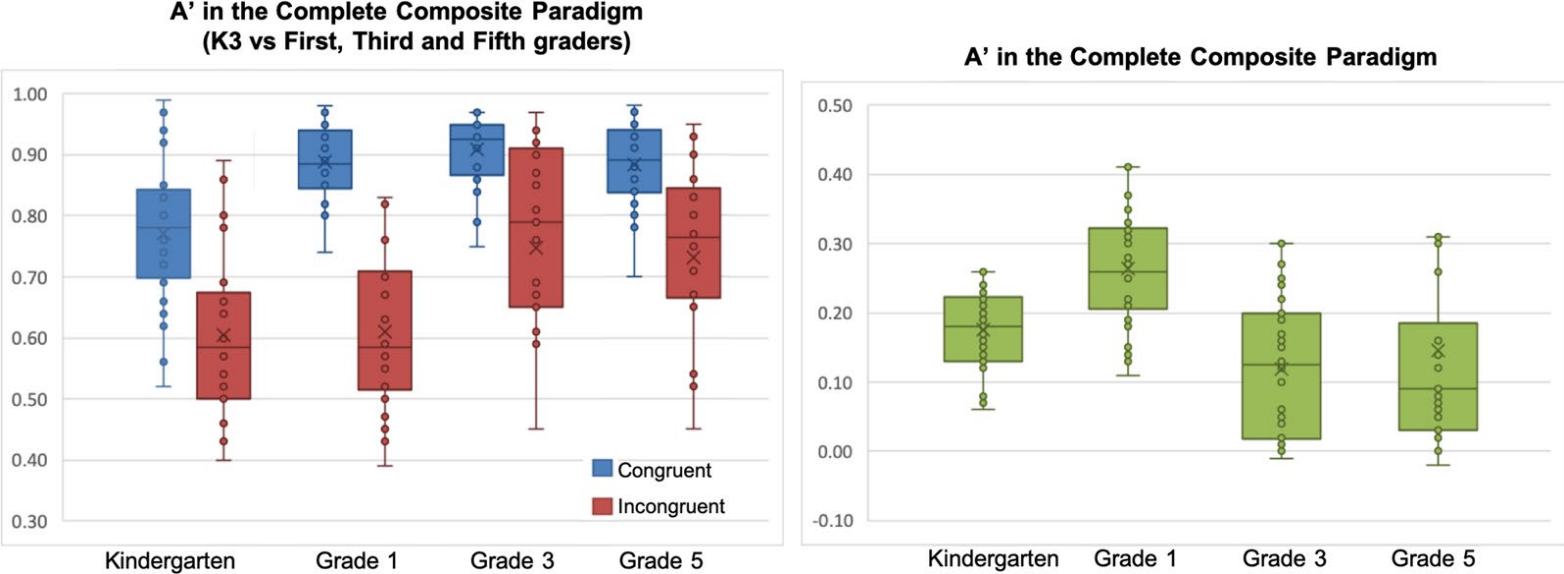
**A**′ of congruent and incongruent trials in the holistic processing task (left) and Holistic **A**′ in Chinese first graders, third graders, fifth graders and kindergartners (right)

Mixed-design ANOVA was used to investigate holistic processing effects (congruency: congruent vs. incongruent trials × grade: K3 vs. Grade 1 vs. Grade 3 vs. Grade 5), with discrimination sensitivity *A*′ as the dependent measure. We found a significant effect of grade, *F*(3, 116) = 10.955, *p* < 0.001, *η*_p_^2^ = 0.221; a significant effect of congruency favouring congruent trials over incongruent ones, *F*(1, 116) = 196.702, *p* < 0.001, *η*_p_^2^ = 0.629, thereby demonstrating holistic processing effects; and an interaction between congruency and grade *F*(3, 116) = 4.543, *p* = 0.005, *η*_p_^2^ = 0.105, with children processed characters with varying levels of congruency effect across grades (Fig. 4). An overall congruency effect was not observed with response time (RT) in the holistic processing task, *F*(1, 116) = 0.342, *p* = 0.56, nor was an interaction effect between congruency and grade, *F*(3, 116) = 0.120, *p* = 0.887. Hence, RT in holistic processing task was not analysed further. This also shows that the congruency effect measured by *A*′ has no accuracy-speed trade-off.^2^

In congruent trials, pairwise post hoc comparisons (Bonferroni corrections: *α* ≤ 0.040) showed that first graders did not differ in *A*′ from either third or fifth graders, respectively *t*(58) = 1.382, *p* = 0.172, and *t*(58) = 0.343, *p* = 0.733; third and fifth graders did not differ significantly either, *t*(58) = 1.689, *p* = 0.10; K3 students had a smaller *A*′ than first graders, *t*(52) = 4.811, *p* < 0.001, *d* = 1.24 and third graders, *t*(58) = 5.820, *p* < 0.001, *d* = 1.50. In incongruent trials, first graders had a smaller *A*′ than third graders, *t*(58) = 3.067, *p* = 0.003, *d* = 0.79, and fifth graders, *t*(58) = 3.066, *p* = 0.003, *d* = 0.79, while third graders had a similar *A*′ as fifth graders, *t*(58) = 0.320, *p* = 0.750; K3 students did not differ from first graders in *A*′, *t*(58) = 0.428, *p* = 0.670, and had a smaller *A*′ than third graders, *t*(58) = 3.262, *p* = 0.002, *d* = 0.84.

In order to further understand the above congruency × grade interaction, an interaction contrast analysis was conducted with reference to procedures suggested by Wiens and Nilsson (2017). With reference to the data presented in Fig. 4, it seems that from K3 to grade 5, the pattern of holistic processing follows an inverted U-shape trend, peaking in first graders. This hypothesis was tested with the (− 1, 3, − 1, − 1) contrast on Holistic *A*′, which was statistically significant, *t*(116) = 3.665, *p* < 0.0001. The other hypotheses on whether Holistic *A*′ peaked in other groups were also tested, and there were no statistical significance for K3 in the (3, − 1, − 1, − 1) contrast, *t*(116) =  − 0.617, *p* = 0.539, third graders in the (− 1, − 1, 3, − 1) contrast, *t*(116) =  − 1.312, *p* = 0.191, nor in fifth graders in the (− 1, − 1, − 1, 3) contrast, *t*(116) =  − 1.737, *p* = 0.085. We also conducted pairwise post hoc *t* tests (Bonferroni corrections: *α* ≤ 0.041) on the *A*′ difference between incongruent and congruent trials (i.e. Holistic *A*′) between children in the four grades: K3 had a smaller Holistic *A*′ than first graders, *t*(50) = 3.474, *p* = 0.001, *d* = 0.90, but K3 children did not differ from third graders, *t*(58) = 0.436, *p* = 0.664, nor fifth graders, *t*(58) = 0.870, *p* = 0.388; first graders had a larger Holistic *A*′ than third graders, *t*(58) = 2.596, *p* = 0.012, *d* = 0.67, and fifth graders, *t*(58) = 3.230, *p* = 0.002, *d* = 0.83, while third graders and fifth graders did not differ in Holistic *A*′, *t*(58) = 0.218, *p* = 0.825. These results suggest a non-monotonic inverted U-shape trajectory of holistic processing of Chinese characters in children learning to read Chinese such that as kindergarten children become emergent readers in first grade, holistic processing first increases and then subsequently decreases in higher grades as writing experience intensifies substantially and analytic processing becomes increasingly important (Fig. 4).

### Pearson’s correlation analysis

Table 2 presents the correlations among age, rapid naming speed, literacy measure, and Holistic *A*′ in the elementary school children. Most of the correlations were statistically significant, except for that between rapid naming speed and Holistic *A*′. Rapid naming speed tasks, although believed to reflect reading processes (Norton & Wolf, 2012), actually measure automatised recognition of overlearned prints, which is sometimes related to reading ability in children (Ho & Lai, 1999; Wolf & Bowers, 1999). Results from Study 1 hence suggest that the developmental changes in holistic processing of Chinese characters (as measured by *A*′) are related to literacy gain in Chinese rather than an improvement in printed symbol (e.g. Arabic numeral) recognition in general.

**Table 2:** Correlations among rapid naming speed, word naming accuracy, dictation accuracy, and holistic *A*′

	Holistic *A*′	Rapid naming speed	Word naming accuracy	Dictation accuracy
Holistic *A*′	–	–	–	–
Rapid naming speed	0.135	–	–	–
Word naming accuracy	− 0.277**	− 0.373**	–	–
Dictation accuracy	− 0.255*	− 0.377**	0.656**	–

*p < 0.05; **p < 0.01

## Study 2

Can the reduced holistic processing from first graders to higher grades observed in Study 1 be explained by something (e.g. better inhibitory control) other than greater Chinese literacy? Study 2 examined holistic processing of Chinese characters in non-Chinese-speaking (NCS) children. In Hong Kong, NCS children are admitted to designated local schools. They should be comparable to the Chinese-speaking children in Study 1 in terms of learning experiences at school. More specifically, their school curriculum was similar to the local Chinese curriculum but delivered in English, so they had minimal instructions on reading and writing Chinese characters. As these children were recruited from the same grades as the Chinese children, we could assume that they were at similar developmental stages. Since these NCS children are novices at recognising Chinese characters, if reduction in holistic processing of Chinese characters is mainly associated with enhanced Chinese literacy, NCS children’s holistic processing of Chinese characters should remain the same as they progress to higher grades, given that their Chinese literacy level would typically remain the same across grades.

### Methods

## Participants

We recruited NCS children from a Hong Kong elementary school. Altogether, 30 first-grade (mean age = 7.00 years, SE = 0.079), 30 third-grade (mean age = 9.33, SE = 0.151), and 22 fifth-grade (mean age = 11.47, SE = 0.198) NCS children participated in Study 2. The smaller sample size in the fifth grade was due to under-enrolment in that school. They learned to read Chinese as an additional language, and the curriculum did not emphasise writing Chinese characters. The main analysis here was the across-grade pattern of holistic processing observed within each group of children (Chinese from Study 1 vs. NCS from Study 2). The two groups of children were all typically developing children matched in age and receiving the same local curriculum (albeit in different languages), and thus they should have comparable general cognitive abilities. All of them had normal or corrected-to-normal vision. The procedures were the same as in Study 1. The measurement on Chinese literacy used in Study 1 was not used for NCS children due to floor effects found in pilot testing, which was understandable as these children were not studying the local Chinese curriculum.

### Results

## Holistic processing

**Fig. 5:**
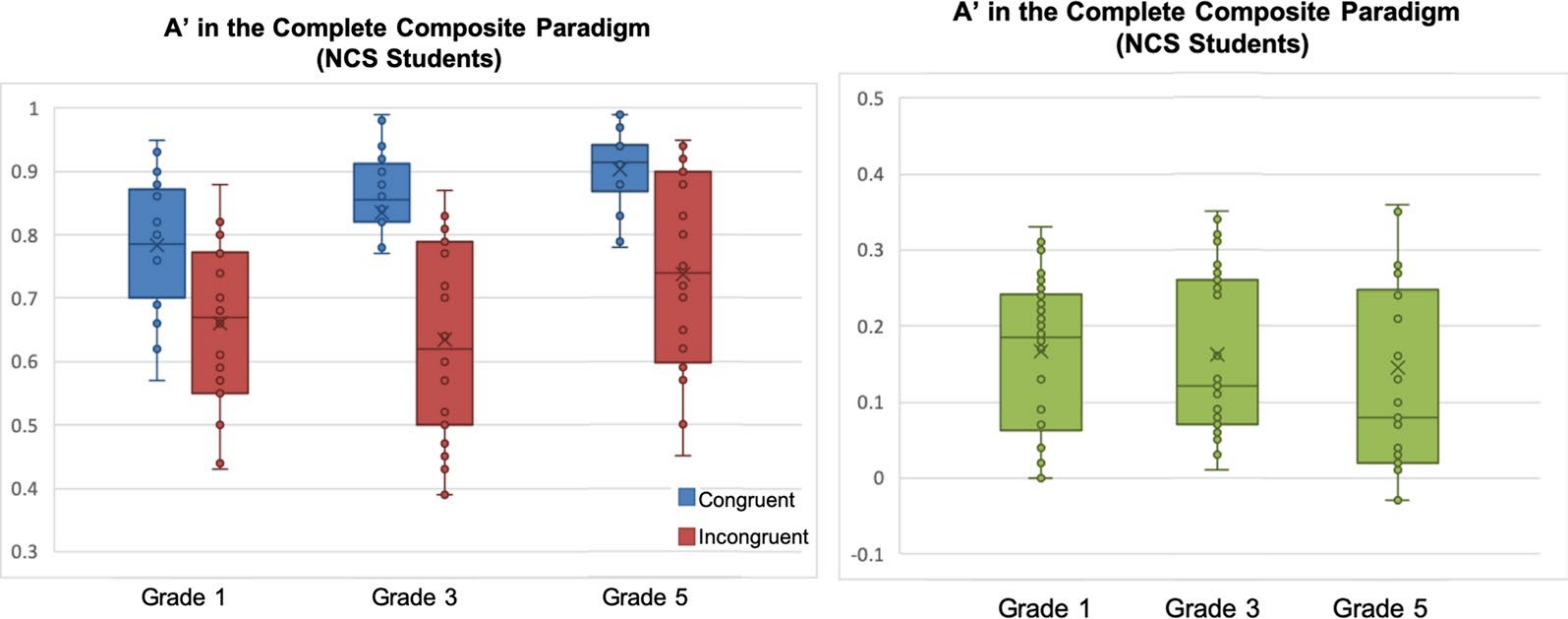
**A**′ of congruent and incongruent trials (left) and Holistic **A**′ in for non-Chinese-speaking first, third and fifth graders in the holistic processing task (right)

Mixed-design ANOVA was used to examine holistic processing effects (congruency: congruent vs. incongruent trials × grade: Grade 1 vs. 3 vs. 5), again with discrimination sensitivity *A*′ as the dependent measure. We found a significant effect of grade favouring higher grades, *F*(2, 79) = 15.496, *p* < 0.001, *η*_p_^2^ = 0.282. There was a significant effect of congruency, *F*(1, 79) = 66.244, *p* < 0.001, *η*_p_^2^ = 0.456; across grades. Importantly, no interaction was found between congruency and grade, *F*(2, 79) = 0.780, n.s.*,* suggesting that these NCS children processed characters with the same level of congruency effect across grades (Fig. 5), and hence the holistic processing effects did not change significantly across grades.

## Chinese versus non-Chinese-speaking students

NCS children did not show reduced holistic processing in higher grades in Study 2, but it remained unclear whether their holistic processing was similar to Chinese-speaking children with limited reading and writing experience. That is, did these NCS children show similar level of holistic processing as Chinese first graders reading Chinese characters? We therefore compared NCS first graders with CS first graders (Study 1).^3^

Specifically, we ran a mixed-design ANOVA with congruency and grade-group combination as the independent variables (congruency: congruent vs. incongruent trials × grade-group combination: NCS Grade 1 vs. CS Grade 1 vs. CS Grade 3 vs. CS Grade 5). We found a main effect of grade-group combination in the predicted direction, *F*(3, 116) = 20.018, *p* < 0.001, *η*_p_^2^ = 0.341, and a main effect of congruency, *F*(1, 116) = 138.86, *p* < 0.001, *η*_p_^2^ = 0.544. We also found a significant interaction between group-grade combination and congruency, *F*(3, 116) = 2.993, *p* = 0.03, *η*_p_^2^ = 0.072.

**Fig. 6:**
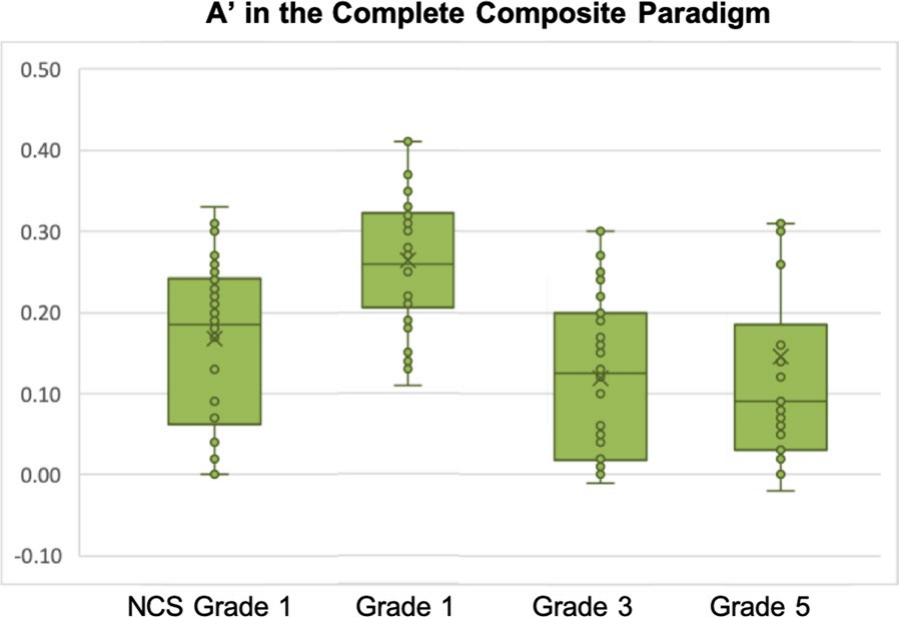
Holistic **A**′ in Chinese first graders, third graders, fifth graders and NCS first graders

In order to further understand the above congruency × group-grade interaction, an interaction contrast analysis was also conducted. With reference to the data presented in Fig. 6, it seems that from NCS Grade 1 to CS grade 5, the pattern of holistic processing also follows an inverted U-shape trend, peaking in CS first graders. This hypothesis was tested with the (− 1, 3, − 1, − 1) contrast on Holistic *A*′, which was statistically significant, *t*(116) = 3.226, *p* = 0.002. The other hypotheses on whether Holistic *A*′ peaked in other groups were also tested, and there was no statistical significance for NCS Grade 1 in the (3, − 1, − 1, − 1) contrast, *t*(116) =  − 0.792, *p* = 0.430, CS third graders in the (− 1, − 1, 3, − 1) contrast, *t*(116) =  − 1.035, *p* = 0.303, nor in CS fifth graders in the (− 1, − 1, − 1, 3) contrast, *t*(116) =  − 1.399, *p* = 0.164. Post hoc *t* tests (Bonferroni corrections: *α* ≤ 0.048) on Holistic *A*′ showed that Chinese first graders were more holistic than NCS first graders, *t*(58) = 2.522, *p* = 0.014, *d* = 0.65, whereas the NCS first graders had similar Holistic *A*′ as Chinese third graders, *t*(58) = 0.134, *p* = 0.894, and fifth graders, *t*(58) = 0.374, *p* = 0.71. These effects suggested that the reduction in holistic processing observed in Chinese-speaking children as they progressed to from first to third grade was mainly due to a stronger holistic processing effect in Chinese first graders when compared with NCS children (Fig. 6).

## Discussion

These two studies set out to investigate the development of holistic processing of Chinese characters in children. Here, we showed that:

1. Chinese-speaking first graders who had learned the Chinese language in a mainstream kindergarten and continued in elementary school exhibited stronger holistic processing in Chinese character processing than either non-Chinese-speaking (NCS) children in English-language elementary school or final-year Chinese-speaking kindergarteners, suggesting an initial increase in holistic processing as children learn to read Chinese characters in mainstream Chinese-language elementary schools.
2. When Chinese-speaking children in mainstream Chinese-language schools progressed to higher grades, they processed Chinese characters less holistically than first graders. This developmental difference was likely a result of enhanced Chinese literacy rather than age or a general improvement in automatised printed symbol (e.g. Arabic numeral) recognition.

### Holistic processing as a consistent expertise marker in object recognition

Holistic processing has been identified as an expertise marker for general subordinate-level object recognition (Bukach et al., 2006; Wong et al., 2009). Comparing holistic processing of Chinese characters in Chinese-speaking and non-Chinese-speaking first graders, our results likewise suggest that holistic processing is an expertise marker for Chinese character recognition at the initial stages of learning to read in Chinese. More specifically, the main difference between the trends observed from Chinese-speaking and NCS elementary school students came from first graders—Chinese first graders processed Chinese characters more holistically than NCS first graders. The Chinese first graders in our study also had much higher Chinese proficiency than NCS first graders (who can be considered novices at recognising Chinese characters).

The stronger holistic processing effect in Chinese-speaking first graders compared with NCS first graders is likely a result of the former’s emergent Chinese literacy skills acquired in Chinese-language kindergarten and part of first grade: Chinese-speaking first graders showed stronger holistic processing of Chinese characters than final-year Chinese-speaking kindergarteners, suggesting that increasing character recognition experience in emergent readers is associated with an increase in holistic processing.

### Non-monotonic trend of development in holistic processing

All the results just described were consistent with Tso et al.’s (2014) speculation about the development of HP for Chinese characters: HP increases when novices start learning to read in Chinese, then decreases as they become experienced in writing Chinese characters. The rise and fall in HP comes quite rapidly—a steep rise from final-year kindergarten to first grade and then a steep drop from first grade to third grade in elementary school. These results point to rather rapid expertise development in Chinese character processing.

The Chinese first graders’ Chinese literacy experiences were most likely not as rich as the third and fifth graders’, and hence they still perceived Chinese characters rather holistically. Hsiao and Cottrell (2009) suggested that expert Chinese readers recognised Chinese characters with a reduced holistic processing as compared with novices, which tells only part of the story—the descending arm of the hypothesised non-monotonic inverted U-shape developmental trend. By contrast, Tso et al.’s (2014) study pointed to the rising arm of the hypothesised trend, showing an increase in holistic processing of Chinese characters in the initial stage of developing expertise in Chinese character recognition—as exemplified by emergent readers with limited writing experience who were international-school students learning Chinese as a non-native language. Through directly examining the relationship between holistic processing of Chinese characters and children’s literacy experiences, here we put two halves of the picture together, demonstrating an inverted U-shape developmental trend of holistic processing of Chinese characters. Perhaps, writing experiences in upper grades helps children to analyse the orthographic structures of Chinese characters, resulting in reduced holistic processing (i.e. more analytical)—this in turn can facilitate character recognition, especially in discriminating the increasingly similar-looking characters as print vocabulary grows.

Converging evidence suggests an analogous relation between face and word recognition in human cognition and brain (e.g. Cao et al., 2019; Dehaene et al., 2010, 2015). Visual similarities between human faces are high; likewise, there is a high degree of visual similarity between Chinese characters (Liu et al., 2016). Here, we postulate that an initial increase in holistic processing for both Chinese characters and faces is necessary for discrimination and individualization of each stimulus. Hence, this inverted-U trend in the study points to the “first more holistic, then more analytic” processing in visual expertise acquisition by making an analogy to face recognition in the case of a reduced holistic processing of faces in artists with face-drawing experiences when compared with ordinary observers (Galmar et al., 2014; Hsiao & Galmar, 2016; Hsiao et al., 2021; Zhou et al., 2012). Although global/configural information was consistently reported to be important for face recognition (e.g. Bartlett & Searcy, 1993; Leder & Bruce, 1998), recent studies suggested that both local/featural and global/configural information plays crucial although different roles (e.g. Burton et al., 2015; Cabeza & Kato, 2000; Chan et al., 2018; Chuk et al., 2017a, b). Intriguingly, computer modelling studies of automatised face recognition showed that algorithms performed the best with both local and global representations of faces, mapping onto analytic and holistic processing, respectively (Bonnen et al., 2013; Ding et al., 2010). The current series of studies showed that children may initially develop a holistic/global representation of Chinese character as their character lexicon expanded through the transition from kindergarten to elementary school, which helped them discriminate between different overall character structures and recognise them efficiently. As the children progressed to upper grades, local attention to character components and stroke was necessary as they write by hand, and learn to discriminate similar-looking characters that become more common. Together, these findings suggested that active retrieval of both global and local character representations are optimal for expert Chinese character recognition.

Our findings are also consistent with the finding that learning to read in Chinese may enhance visual spatial skills in Children (McBride-Chang et al., 2011a, b). Heavy “look-and-say” instructions are involved in the local Hong Kong Chinese literacy classes (Holm & Dodd, 1996; Huang & Hanley, 1995), and hence sensitivity to the visual logographic properties is crucial for reading. Our results suggest that both the initial increase in holistic processing and the development of analytic processing of Chinese characters are crucial for the development of Chinese literacy. It is perhaps not surprising that visuo-spatial deficits typically marked the core cognitive profile for dyslexia in Chinese children (Siok et al., 2009), and that students with reading difficulties had deficits in selectively attending to parts, leading to increased holistic processing (Tso et al., 2020).

Previous studies reported an increase in orthographic awareness with grade (Ho et al., 2003), and here we offer an information processing account of the perceptual processing change that may occur in children as they gain in literacy. However, since writing practice is implemented regularly and rather intensely for the language curriculum in Hong Kong, our studies could not tease apart the unique influence of reading and writing on holistic processing. Nonetheless, holistic processing did not correlate significantly with rapid naming speed, suggesting that holistic processing in character recognition is associated with an increase in Chinese literacy and not a general improvement in printed symbol (e.g. Arabic numeral) recognition. Moreover, Study 1 shows that reduction in holistic processing across grade coincided with a decrease in the discrepancy between word naming and writing, consistent with Tso et al.’s, (2014) findings. Future studies can investigate the holistic processing developmental pattern in Chinese character recognition where reading and character-writing are more easily teased apart—with students from international schools or overseas Chinese children who often learned to “write” Chinese characters with computer software that converts phonic alphabet input (e.g. pinyin) or speech input into characters for selection relying on visual recognition (Tso et al., 2014). Future studies can also examine the relationship between the developmental trend of holistic processing and Chinese character frequency to better understand the cognitive mechanism when readers recognise Chinese characters of different levels of familiarity.

In contrast to Chinese character reading, analytic processing of letter-sound properties in English words is shown to mark the initial learning stages in preschool native and second-language English learners (McBride-Chang & Treiman, 2003). Thus, the developmental trend of holistic processing in English word reading deserves future investigations as it may be different from that in Chinese character reading due to the requirement of letter-sound mapping. The findings from this paper are also consistent with Agarwal et al.’s (2019) study that showed an increased compositionality in readers of Telugu and Malayalam (Indian languages of alphabetic writing systems) when compared with non-readers. Indeed, holistic processing as an expertise marker is shown to have limited transfer effects across different expertise domains (e.g. Liu et al., 2016).

Note that paradigms in which upside-down stimuli are shown to impair recognition performance, i.e. inversion effect, have been suggested to illustrate holistic/configural processing (Sekuler et al., 2004). However, this paper argues that inversion effect demonstrates novelty effect in perceptual learning rather than the type of holistic processing illustrated in this paper (i.e. the obligatory perceptual integration of both featural and configural information of visual objects; see Maurer et al., 2002). Since inversion effect has been demonstrated as an expertise marker of Chinese character recognition in typical readers (i.e. this effect is less susceptible to writing experiences), perhaps there will be a gradual increase in this effect across grades as the children improve in Chinese reading performance (Kao et al., 2010). This speculation can be examined in future studies.

### Limitations

Note that here we used the same materials to measure literacy development across grades. While this design could directly compare literacy level difference across grades, it may not adequately reflect individual differences in literacy performance within each grade. Future work may examine the relationship between individual difference in holistic processing and literacy performance within a grade using a more sensitive measure. Nevertheless, by using the same materials across grades, we found a discrepancy between word naming and dictation across grades, consistent with prior studies that suggested a disconnection between reading and writing skills (Tso et al., 2014, 2020). Future studies may explore this discrepancy and its association with holistic processing.

In addition, K3 students had smaller *A*′ than both first and third graders in congruent trials but had smaller Holistic *A*′ than first graders and a similar level of Holistic *A*′ to third graders, suggesting that the holistic processing pattern found in Chinese children—the non-monotonic trend from kindergarteners to third graders—could not simply be explained by a ceiling effect in congruent trials. Note also that the *A*′ in congruent and incongruent trials in K3 were both above chance level, *t*(29) = 12.716, *p* < 0.001, and *t*(29) = 3.440, *p* = 0.002, respectively, suggesting that the effect was unlikely to be due to a floor effect. Moreover, the finding from congruent trials suggested that reading experience facilitates feature integration in congruent trials as children progressed from K3 to Grade 1. Here, we aimed to examine the changes in cognitive processing pattern of Chinese character recognition when children of different literacy levels were presented with the same Chinese character stimuli under the same experimental conditions. Future work can adjust the research design when examining holistic processing of Chinese characters in children at a particular literacy level. Moreover, the non-monotonic pattern of holistic processing observed in this study mainly arises from the comparison of Chinese first and third graders with NCS and kindergarteners. The measurement on Chinese literacy used in Study 1 was not used for kindergarten and NCS children due to floor effects shown in our pilot. At the moment, there is a lack of standardised Chinese literacy tests to assess preschoolers and second-language Chinese students in Hong Kong. Follow-up studies should indeed validate a standard measurement to measure literacy skills across the groups from our study.

It is also noted that the conclusion from this paper is based on cross-sectional comparisons. Future studies may explore possible longitudinal comparison of HP by following the developmental trajectory of children’s literacy development. However, it is noted that cautions be taken in order to reduce the possibility of practice effects in the composite paradigm.

## Conclusion

Here, we have discovered a non-monotonic trend of holistic processing in children learning to read Chinese: emergent readers using more holistic processing of Chinese characters with increasing character recognition experience, followed by reduced holistic processing as they reached upper grades with more extensive Chinese reading and writing experiences. This reduction in holistic processing should facilitate Chinese word reading and writing as children are required to selectively attend to character components more during these processes. Our findings thus provide a perceptual processing account of the close relationship between reading and writing performance previously reported in the literature.

In addition, our results offer a unifying and theoretically more satisfying account for mastering visual recognition across domains. Specifically, an increase in holistic processing may be an expertise marker of visual object recognition (including Chinese character recognition) at an initial stage, which marks the development of holistic processing skills, whereas expertise development may require more analytic processing skills at a later stage, leading to reduced holistic processing. Both holistic and analytic processing skills seem to be important in mastering visual recognition.

## Data Availability

Since this study is ongoing, the data sets of the current study are available from the corresponding author on reasonable request.

## Footnotes

1 The participants in Study 1 were from another project that involved a larger sample size, and they were resampled to match with the sample size of each group in Study 2 of around thirty participants. Based on the power analysis with G*Power, the required sample size in each group to detect a medium effect in a repeated-measures ANOVA is twenty-two, and hence thirty participants in each group were adequate.

2 Similar results were found using *d*'.

3 A 2 × 3 × 2 repeated-measures ANOVA (congruency × grade × group: NCS vs. Chinese) revealed a main effect of grade, *F*(2, 166) = 19.093, *p* < .001, *η*_p_^2^ = .187, a main effect of congruency, *F*(1, 166) = 178.296, *p* < .001, *η*_p_^2^ = .510, a main effect of group, *F*(1, 166) = 11.028, *p* = .001, *η*_p_^2^ = .062, and a congruency × grade interaction, *F*(2, 166) = 3.768, *p* < .025, *η*_p_^2^ = .043. There was no group × congruency interaction, *F*(1, 166) = .733, *p* = .393, or group × grade × congruency interaction, *F*(2, 166) = 1.587, *p* = .208. Note that a limitation of this study is the small sample size in each grade and a smaller number of NCS fifth graders, which may hinder the detection of a true group × congruency × grade effects. However, the decision for the analyses for the analysis in Study 2 was made post hoc to better highlight the hypothesised non-monotonic trend of holistic processing. Since the robust change in holistic processing was only observed from first to third graders with Chinese-speaking children but not from third to fifth grade in Study 1, the following analysis with only first and third graders was also conducted for a more focused analysis, which revealed a main effect of grade, *F*(1, 116) = 18.233, *p* < .001, *η*_p_^2^ = .136, a main effect of congruency, *F*(1, 166) = 178.296, *p* < .001, *η*_p_^2^ = .510, and a main effect of group, *F*(1, 116) = 150.747, *p* < .001, *η*_p_^2^ = .565. There was a marginal congruency × grade interaction, *F*(1, 116) = 2.958, *p* = .088, *η*_p_^2^ = .025, and a group × grade × congruency interaction, *F*(1, 116) = 3.027, *p* = .085, *η*_p_^2^ = .025 (there was no group × congruency interaction, *F*(1, 116) = .648, *p* = .42). Further post hoc analyses showed no difference in Holistic A’ between Chinese and NCS students in grade 3, *t*(58) = .683, *p* = .498, and in grade 5, *t*(58) = .340, *p* = .683. It seems that the subtle difference in the developmental pattern of holistic processing between Chinese and NCS students lied in first graders.
